# Exploring advanced clinical practitioner perspectives on training, role identity and competence: a qualitative study

**DOI:** 10.1186/s12912-024-01843-x

**Published:** 2024-03-18

**Authors:** Maxine Kuczawski, Suzanne Ablard, Fiona Sampson, Susan Croft, Joanna Sutton-Klein, Suzanne Mason

**Affiliations:** 1 Centre for Urgent and Emergency Care Research (CURE), Sheffield Centre for Health and Related Research (ScHARR), The University of Sheffield, S1 4DA Sheffield, UK; 2 Emergency Department, Northern General Hospital, S5 7AU Sheffield, UK; 3 Manchester Royal Infirmary, Oxford Rd, M13 9WL Manchester, UK

**Keywords:** Advanced clinical practitioner, Qualitative, Role identity, Transition

## Abstract

**Background:**

Advanced Clinical Practitioners (ACPs) are a new role that have been established to address gaps and support the existing medical workforce in an effort to help reduce increasing pressures on NHS services. ACPs have the potential to practice at a similar level to mid-grade medical staff, for example independently undertaking assessments, requesting and interpreting investigations, and diagnosing and discharging patients. These roles have been shown to improve both service outcomes and quality of patient care. However, there is currently no widespread formalised standard of training within the UK resulting in variations in the training experiences and clinical capabilities of ACPs. We sought to explore the training experiences of ACPs as well as their views on role identity and future development of the role.

**Methods:**

Five online focus groups were conducted between March and May 2021 with trainee and qualified advanced clinical practitioners working in a range of healthcare settings, in the North of England. The focus groups aimed to explore the experiences of undertaking ACP training including supervision, gaining competence, role identity and career progression. Thematic analysis of the focus group transcripts was performed, informed by grounded theory principles.

**Results:**

Fourteen advanced clinical practitioners participated. Analysis revealed that training was influenced by internal and external perceptions of the role, often acting as barriers, with structural aspects being significant contributory factors. Key themes identified (1) clinical training lacked structure and support, negatively impacting progress, (2) existing knowledge and experience acted as both an enabler and inhibitor, with implications for confidence, (3) the role and responsibilities are poorly understood by both advanced clinical practitioners and the wider medical profession and (4) advanced clinical practitioners recognised the value and importance of the role but felt changes were necessary, to provide security and sustainability.

**Conclusions:**

Appropriate structure and support are crucial throughout the training process to enable staff to have a smooth transition to advanced level, ensuring they obtain the necessary confidence and competence. Structural changes and knowledge brokering are essential, particularly in relation to role clarity and its responsibilities, sufficient allocated time to learn and practice, role accreditation and continuous appropriate supervision.

**Supplementary Information:**

The online version contains supplementary material available at 10.1186/s12912-024-01843-x.

## Introduction

Advanced Clinical Practitioners (ACPs) are a relatively new role in the National Health Service (NHS), introduced to address the increasing complexity of healthcare needs and the growing demand for skilled professionals. They are becoming increasingly embedded within a wide range of NHS healthcare settings spanning community services, mental health wards and hospitals. ACPs play a vital role in expanding the scope of practice within healthcare teams, take on more advanced and complex levels of clinical work, including tasks historically carried out by doctors, with an aim to help alleviate the strain on medical professionals and enhance the efficiency of healthcare delivery. Their integration into the workforce has been shown to enhance patient care by providing timely access to high-quality services while also fostering interdisciplinary collaboration [1]. Studies have shown that ACPs contribute to improved patient outcomes, increased patient satisfaction, and cost-effective healthcare delivery [2, 3]. Additionally, their presence supports the development of junior staff by providing mentorship and guidance, thus ensuring a sustainable healthcare workforce for the future [1, 4]. As non-medical healthcare professionals, ACPS are required to undertake further education (Masters degree) and extended training in specific clinical areas such as nursing, pharmacy, or allied health professions to qualify as an ACP. According to the multi-professional framework advanced clinical practice, this training is underpinned by four pillars: clinical practice, leadership and management, education, and research [5]. However, there is wide variability in this practice and training of ACPs across the UK [6].

## Background

Recent years have seen attempts to standardise the training and practice of ACPs. A framework for advanced clinical practice in England was authored in 2017 by Health Education England (HEE) (NHS England) [5], which set out standards for advanced clinical practice. Within this framework, advanced clinical practitioners should be able to deliver care with a high degree of autonomy and undertake complex decision making. The knowledge and skills should be underpinned by a Master’s level award (or equivalent) that incorporates the Four Pillars of Practice: Clinical Practice, Leadership and Management, Education and Research [5]. In 2020, The Centre for Advancing Practice (https://advanced-practice.hee.nhs.uk/) began accrediting some of the many advanced clinical practice Masters programmes available in the UK, which it deemed to have met the standards laid out in HEE’s framework [7]. This process of defining common standards remains in its early stages, and there remains little research on ACP training programmes and their structures or governance. In a further effort to improve and standardise advanced clinical practice, The Centre for Advancing Practice additionally created guidance on workplace supervision for ACPs, noting the crucial need for high-quality supervision [8]. The Nursing and Midwifery Council (NMC) published their 2020-25 corporate strategy also in 2020, and committed to explore the need for regulation in a comprehensive review of advanced nurse practice [9]. The review is still in progress but research undertaken in the early stages by The Nuffield Trust and BritainThinks as part of the review reported inconsistency in definitions, outcomes, standards of education and proficiency in advance practice [10], and support for regulation by health professionals [11]. Despite calls for improvements in the supervision of ACPs, there has been limited research in this area.

Studies have shown that ACPs have historically struggled with the transition from their previous career to their advanced practice roles [12–15]. The challenges of the transition have been exacerbated by a lack of clear professional identity for ACPs, which has been noted to be a source of tension and confusion, impacting on training, development and ultimately patient safety [6, 16–20]. Recognising the importance of successful integration into the workforce will help ACPs to realise their full potential [21, 22], impacting on role satisfaction [23], staff retention [24] and ultimately, building a more sustainable workforce.

As efforts to standardise and develop the ACP role continue, ACPs are becoming more widespread within the NHS. The proliferation of ACPs brings a need for a better understanding of all aspects of ACP training, both during and after qualification. We sought to explore the training experiences of ACPs with the aim of informing future models of education and support.

### Theoretical framework

The theoretical framework of this study is based on the theories of Bourdieu, particularly his concept of Habitus [25], as it offers a valuable lens for examining the multifaceted identities, roles, and positionalities of ACPs. Habitus, ingrained dispositions and cultural knowledge shaped by social experiences, acts as a bridge between individual practitioners and the complex healthcare field they navigate. It influences how ACPs perceive and enact their roles, shaped by their educational background, professional training, and prior clinical experiences. Furthermore, Habitus interacts with the “field,” the social space within which ACPs operate, characterized by power dynamics, established hierarchies, and competing ideologies. This interaction influences the capital, both symbolic and material, that ACPs possess and wield within the field. Through this lens, we can understand how ACPs negotiate complex power dynamics within the healthcare system, navigate tensions between professional autonomy and institutional constraints, and ultimately construct their own sense of meaning and purpose within their evolving roles. By analyzing these interactions between Habitus, field, and capital, Bourdieu’s framework offers a rich and nuanced understanding of the experiences and challenges faced by ACPs, paving the way for further research and dialogue on optimizing their practice and impact.

### The study

#### Aim

We sought to explore the training experiences of ACPs as well as their perceptions on role identity, gaining clinical competency and future development of the role.

## Methods

### Design

This exploratory study used a qualitative design to conduct focus groups with a purposive sample of ACPs currently working in South Yorkshire and Bassetlaw in the North of England. In 2022 there were 585 trainee ACPs and approximately 1200 qualified ACPs working in this region.

### Participants

Qualified ACPs or trainee ACPs that have completed at least 1 year’s full time equivalent of Advanced Care Practitioners clinical training, and currently work in this role within either Mental Health, Community or Secondary Care within the South Yorkshire and Bassetlaw region. It was felt 12-month minimum training experience would ensure trainees were sufficiently embedded in the clinical and educational programmes.

### Recruitment

The NHS England Regional Faculty for Advancing Practice– North East and Yorkshire (FACP-NEY) acted as gatekeepers for the recruitment, contacting all qualified and trainee Advanced Care Practitioners working in the region with an invitation to participate by email. The email included a brief outline of the study, dates and times of the focus groups, details of an incentive payment of £30 for participation, a participant information sheet and, a web link to a short online questionnaire and contact details form. Additionally, the study was also advertised on social media platforms (Twitter, Facebook), with those who expressed an interest sent the same study invitation email, documentation and web link. Recruitment was open between February and May 2021, with one reminder email sent from the FACP-NEY during this time.

ACPs that wished to participate in the study were required to complete the short online questionnaire built using the survey tool, Qualtrics (www.qualtrics.com). After confirming eligibility, basic details were recorded about the participant and their ACP training including name, contact details, gender, age group, ethnicity, length of experience in the ACP role, supervision routine, portfolio status and supernumerary time. A variety of dates and times (morning, afternoon and evening) were provided for the focus groups to maximise recruitment, and participants were asked to indicate their preference. A total of 14 participants took part across five focus groups.

### Data collection

Focus groups took place online using the Google Meets platform, with a maximum of 3 participants per group. To ensure participants were confident in using the Google Meets platform, the focus group began with an overview of the main functions and how to use them, for example clicking the ‘hand-up’ icon to indicate a wish to speak and chat facility. A focus group schedule was designed and used to guide the discussion similar to that used by Macnaghten and Jacobs (1997) [26] with an emphasis on each topic followed by discussion amongst the participants. The topics covered included experiences of undertaking ACP training (including gaining competence), role identity and career progression. Data collection was discontinued once it was felt there was no new contributions to the analysis, and there had been full investigation of the developed themes.

Participants provided written informed consent prior to attending the focus group, and consent was also acquired verbally at the start of each focus group. Each focus group was facilitated by one of the two authors (SA and MK), both of whom are experienced qualitative researchers with no clinical background or experience. Google Meets was used to video and audio-record the focus groups. The focus groups were transcribed verbatim by a third party, and quality checked against the recordings for accuracy. The duration of the focus groups was 2 h with a 15-minute comfort break. On completion of the focus groups, participants were sent a £30 shopping voucher to compensate them for their time.

### Data analysis

The data was thematically analysed by three researchers (MK, SA and JSK) following the six-phrase process of Braun and Clarke, commencing with familiarisation of the data and then line by line coding to identify preliminary categories [27, 28]. The data was then ordered and synthesised, combining similar categories and exploring the relationships between them [29]. This process was repeated for three of the five transcripts at which point the main themes and sub-themes were identified forming a test model, this was then applied to the final two transcripts. Following discussion amongst the research team, the main themes and sub-themes were agreed. NVIVO Release 1.3 (QSR International) [30] was used to help organise the data. The Standards for Reporting Qualitative Research (SRQR) checklist was used to report the findings (see Additional file 1).

## Results

The focus groups highlighted significant variability in the training experience of ACPs, dependent on their role and place of work. Table 1 provides an overview of the participant characteristics of each of the focus groups, and an overview of the overarching themes and sub-themes that were developed are displayed in Table 2.

**Table 1:** Focus group participant characteristics

Participants	Focus group				
	1	2	3	4	5
**Total**	3	3	2	3	3
**Sex: Female**	3	2	2	3	3
**Age group (years)**					
*25–34*	-	-	-	1	2
*35–44*	2	2	1	-	1
*45–54*	1	1	1	2	-
**Ethnic origin**					
*White (British/ Irish/ Other)*	2	3	1	3	3
*Mixed*	1	-	1	-	-
**ACP status**					
*Training*	3	1	1	3	2
*Qualified*	-	2	1	-	1
**Employed**					
*Primary/ Community care*	-	1	-	2	1
*Secondary care* *(including Mental Health Trust)*	3	2	2	1	2

**Table 2:** Overview of the themes and sub-themes from the focus group

Theme	Sub-themes
Clinical training lacked structure and support	*Academic: well-supported, would benefit from being more bespoke.*
	*Clinical: variable, placement issues, lack of formal structure for supervision*
	*Lack of coherence between academic and clinical settings*
	*Clinical supervision and line management*
Existing knowledge and experience appeared to act as both an enabler and inhibitor for ACPs, with implications for confidence	*Pattern recognition, learning backwards*
	*Identifying own knowledge/learning gaps*
	*Competence and confidence*
The ACP role and associated responsibilities are poorly understood by ACPs and the wider medical profession	*Variability across organisations*
	*Lack of awareness and skill utilisation*
	*Struggle to fit in and identify/explain the role*
The ACP role is important, but changes are required to provide security to the role in the future	*Clinical career progression*
	*Barriers to development of role in the future*
	*Accreditation*

### Overarching themes

A number of overarching themes were identified in our analysis that appeared to be strongly linked to role identity. We found the experiences of the ACP training were influenced by internal and external perceptions of the ACP role, often acting as barriers, with structural aspects being significant contributory factors. These findings were revealed in four key themes - lack of structure and support in the clinical training, existing experience and knowledge as enablers and inhibitors to progress with implications for confidence, the poorly understood nature of the ACP role and associated responsibilities, and a need for change to provide security to the ACP role in the future.

### Clinical training lacked structure and support

The data revealed a stark contrast between the academic and clinical training, with clinical training found to be lacking in structure and support. Experiences of the clinical training were often expressed negatively due to the lack of structure which was heavily reliant on supervision and placements. As a result, ACPs often had to take the lead on their training and having to identify their own supervisor(s) and/ or placements was felt to be challenging. Consequently, some ACPs reported they had no dedicated medical supervisor at all. Where supervisors were in place, the quality of supervision varied, from being *ad hoc* (p41) and *chaotic* (p52) to *great (*p53). Some of the supervision issues raised by the ACPs included lack of supervisor knowledge in relation to the ACP training and their required responsibilities, accessibility of supervisor (available time) and little direct clinical oversight. ACPs felt they needed an experienced medical professional as their supervisor, providing similar support and advice to that received by junior doctors.

*We have nursing supervision from the lead community matron who is our line manager, but we do miss that sort of medical supervision (p22, Trainee ACP– Primary care)*.

*I’m line managed by a nurse who is the operational lead for the service. He is the right person, but I don’t go to him for clinical support. It would be nice to have a medical supervisor (p. 41, Trainee ACP– Community care)*.

Good supportive supervision appeared to enhance the ACP training, conversely poor, unsuitable or no supervision was perceived to have a serious negative impact on training and well-being, with suggestions that ACPs had left during training because of it.

*I’ve had free reign over my own training, and planned everything myself, and that’s a positive for me (p41, Trainee ACP– Community care)*.

*So the positives, um, I think the academic and educational supervision’s been, err, accessible and supportive. So we have, um, supervision from [regional] ACP lead,…and then there’s, um, the course unit lead, which she’s there and she’s supportive. So yeah, the academic, err, supervision is good (p52, Trainee ACP– Secondary care)*.

I think, um, something that I haven’t touched upon is, which I realised, so I’ve got a, um, clinical supervisor, she’s a consultant *****, and…the module I’ve just done which is minor illness, you had to do like a learning log, so they had to see you do….a load of things. And it made me laugh cos they turned around and said, look, I haven’t assessed anybody’s abdomen in ten years…. (p53, Trainee ACP– Secondary care)

Similar to supervision, clinical placements were highly valued by the ACPs and recognised as an important part of the training to achieve competence and consolidate their academic learning. All of the ACPs reported obstacles in organising and undertaking such placements, with those working in the community or mental health facing particular difficulties due to placements needing to be in a different clinical setting to where they worked. Competition with other trainees, the need to ‘*beg*’ (p7) and insufficient time from trainers were highlighted as ongoing problems. Conflict with junior doctors was also described as a competition for training opportunities.

*Completely unsupported by the Trust because they just weren’t set up for it, there was no one leading on it, there were no one for us to contact really to talk. And then, like you said, I got my placements from begging on a, on a forum on Facebook and a nurse set me up (p16, Trainee ACP– Secondary care)*.

*To kind of fulfil the module requirements, it was pretty much, for minor illness basically phoning up GP surgeries, practice nurses, beg stealing and borrowing, you know, begging people can you help me out, to try and get the amount of hours that you needed (p7, Trainee ACP– Secondary care)*.

*But sometimes, it’s a little bit of a fight to get to what you need when you need because there’s so many junior doctors that also need that same training. So, there are occasions where you have to sort of step up and say we are training the same as these guys, we also need to be able to have these opportunities and you kind of have to have a little bit of a voice to say, we’re here (p17, Trainee ACP– Secondary care)*.

In contrast to the clinical training, the academic learning followed a traditional format of taught lessons which ACPs felt covered a wide breadth of knowledge. There was some feeling that modules might have been more useful if they had been tailored towards individuals’ specialisms such as mental health or physiotherapy, however on the whole it was described as a positive learning experience with good supportive academic supervision.

*I found the dissection labs quite alien but they have really helped to develop my practice (p24, Qualified ACP– Secondary care)*.

*It feels a lot like there’s university, which is one day a week, and you do that, and it’s really supportive, and I’ve made some really good friends there, and everybody supports each other. But then at work, it’s a bit of a try and find your own way (p53, Trainee ACP– Secondary care)*.

ACPs did describe the two learning environments (clinical and academic) as disconnected, separate and discrete, even though the ACP training is a combination of academic and clinical learning.

*From the course point of view it’s pretty straightforward but it’s marrying that up with the expectations of the employer. Willingness of the employer to be able to give you the time you need to do what you need to do (p. 38, Qualified ACP– Secondary care)*.

*They’d learn something at University (e.g. Cardiology) but there was no way this could be built on within the Trust. They just don’t deal with the physical health side of things (p. 7, Trainee ACP– Community mental health)*.

### Existing knowledge and experience appeared to act as both an enabler and inhibitor for ACPs, with implications for confidence

As existing experienced clinical practitioners, ACPs felt they were able to recognise their knowledge gaps and work quickly towards filling them, however the training approach also led to declines in confidence when deficiencies in knowledge and skills were highlighted. ACPs reported learning ‘backwards’ compared to junior doctors, using pattern recognition rather than pathology as a starting point, for example, being able to identify the treatment based on a diagnosis, but not necessarily knowing how the diagnosis was made originally. Not being able to adequately answer questions sufficiently on such subjects when tested by clinicians, and as experienced clinical practitioners, ACPs perceived themselves as lacking competence with a subsequent drop in confidence.

*ACPs are trained ‘bottom-up’– we learn pattern recognition and then work our way back, whereas doctors know the diseases better (p41, Trainee ACP– Community care)*.

*I think about cases backwards compared to doctors– as they think about pathology first and then build on that (p9, Trainee ACP– Primary care)*.

A comparison between the clinical training processes of junior doctors and ACPs was a common discussion between ACPs with suggestions that it would be more beneficial if ACPs were recognised in a similar manner to junior doctors. For example, ACPs felt they should not be ‘counted in the nursing numbers’ when working on a ward, and as a consequence should not be expected to undertake a dual role of managing a nursing shift and practicing as an advanced practitioner:-

*So say for example, you’re sat with somebody talking about their prescription and trying, you know, looking to see if there needs to be a change made, and then you’ve got other people banging on the door saying, I want to go out on leave, and I need this and I need that, and you’re the nurse in charge and need to be doing that. The people that usually do those jobs, so say for example the doctors in the week, when they’re having those sorts of consultations with people, they’ve not got that stress, the pressure, the disruption and the responsibility of running a nursing shift or a completely other shift. So, us as novices, it just doesn’t make sense to me (p. 52, Trainee ACP– Secondary care)*

ACPs spoke of being unsure of when they had reached clinical competency, and how they would maintain this. They worried that if they were not given sufficient time to practise the new clinical skills, their confidence would decline and that they would ultimately feel unsafe in their clinical practice. ACPs emphasised the importance of having sufficient time to practice new skills and consolidate knowledge, enabling autonomy and confidence building. It was also felt this provided essential opportunities for colleagues to observe progress.

*I’ve got most of my competencies but I still wouldn’t see myself as an expert practitioner (p41, Trainee ACP– Community care)*.

### The ACP role and associated responsibilities are poorly understood by ACPs and the wider medical profession

Exploring the experiences of training and the process of developing clinical competence with ACPs revealed there was a lack of clarity regarding the job role depending on where the ACP worked, and this applied to the ACPs themselves as well as their colleagues. This uncertainty impacted the responsibilities the ACP undertook within the clinical environment, and the expectations on them from the staff that they worked with.

ACPs that worked within the Emergency Department reported that colleagues understood the ACP role and utilised the advanced skillset the ACPs gained as the training progressed. They described feeling fully immersed within the department as an advanced practitioner, yet they were also recognised as being in a transitional stage with appropriately allocated time to undertake the necessary training.

ACPs working in other areas of healthcare such as acute wards, outpatients, mental health and community care discussed a general lack of awareness about the advanced practitioner role by both healthcare staff and patients. It was felt this led to a lack of utilisation of the advanced skills of the ACPs and expectations that the ACP should fulfil multiple job roles, creating feelings of intense pressure and demoralisation. ACPs reported hearing discouraging comments from colleagues about their abilities and felt a need to justify their role. Some ACPs described struggling with how to introduce themselves to both staff and patients, with their uniform described as an important part of their identity and how they were perceived by others. Adding to these external perceptions, ACPs revealed their job description was not necessarily updated to reflect their ACP role and where it was, the job description could be vague further undermining their role identity and leading to feelings of conflict between their original healthcare professional role (e.g., nurse) and working at an advanced level.

*There’s been a lot of ambiguity around the job description for ACPs and trainee ACPs, so that’s left wriggle room for everybody making their own assumptions about what you’re supposed to do and what you should be doing, and therefore you’re pulled into all different things that don’t tie in to on paper in terms of national, regional frameworks……. there’s just pressure on the role being categorised as an extension of the nursing team, and taking on classic nursing tasks, it’s what people are familiar with, it’s what they assume (p52, Trainee ACP– Secondary care)*.

*The challenge is with our role, is the ACP is tagged on to the end of our existing job. So, we have all of our normal nursing duties, we’re bed managers, we triage nurse, we run the hospital. And then you’ve got ACP tagged on the end. (p25, Qualified ACP– Secondary care)*

Inconsistencies in awareness of the role, experience, training and clinical practice were felt to be a reflection of the different professions undertaking ACP training, a lack of standardised job role and unclear expectations. The variation in financial remuneration within and across different organisations for ACPs was also felt to be a contributing factor to these identity issues.

### The ACP role is important, but changes are required to provide security to the role in the future

There was consensus that the combination of experience and advanced skills made the ACP a unique and valued role in the NHS, fulfilling an important gap in patient care. ACPs reported uncertainty about their future in the role, and the need for change structurally to ensure the ACP role has a future. Accreditation was felt to be necessary as this would legitimise the ACP role and apply some professional control in respect to the role title. ACPs viewed this as an existing issue with ‘advanced’ used by a multitude of health professions that have not undertaken the accredited training.

*I kind of feel that, certainly as an ACP title, it should be some sort of standardised title, and then people would probably understand it a little bit more. I think our colleagues would understand it, and I think you won’t get so much resistance, from some medical colleagues, maybe, if people were sort, if it were a bit more regulated. I mean, if there were talking about credentialing and looking at a directory for ACPs anyway, it should be a registered regulated title (p54, Qualified ACP– Primary care)*.

*I think everybody should be under the same governing body and there should be a bit of standardised, training placement (p41, Trainee ACP– Community care)*.

As well as increased knowledge and skills, ACPs discussed the additional benefits of the training including the broad range of opportunities offered both during and after the training, and the potential boost in future prospects. A key attraction to the ACP training route that was repeatedly highlighted was the fact that it offers career progression whilst maintaining clinical responsibilities, progressing through more traditional routes into a managerial role appears to involve considerably less clinical duties and contact with patients. However, there was also some feelings of insecurity regarding the future of the ACP role because of the general lack of awareness of how ACPs fitted and could contribute to the NHS. It was felt that the deficiency in formal structure for the ACP role contributed to this; ensuring job descriptions existed and reflected the responsibilities of the role, and there was a structure for career progression was proposed as a good starting point to improve understanding amongst staff.

*In terms of where I see myself in five to ten years’ time, I’m not sure, it depends how that organisation I work for pans out, because…. I won’t be sat here in five years’ time saying the same stuff. If it’s still the same I won’t be there, I will have gone somewhere else cos there are places that fulfil the role (p52, Trainee ACP– Secondary care)*.

*I don’t see much career progression within ACPs other than to become a lead ACP and there is nothing to define progression within that role from a banding point of view (p1, Trainee ACP– Secondary care)*.

On the whole, the ACPs felt the role had great future potential but this was often caveated, that changes were needed in formalisation of the training and particularly, wider recognition of the role and its responsibilities. Without these changes, a number of ACPs felt they would not be in the ACP role in 5 years’ time.

*The one thing that I do know is that I love the job, I love the role (p38, Qualified ACP– Secondary care)*.

## Discussion

This qualitative study collected the perspectives of 14 ACPs from different specialties and at different stages of their career. The findings suggest that ACPs continue to face significant barriers, undermining their development, transition and integration into the healthcare workforce.

ACPs described a number of challenges experienced in their training within the clinical environment, notably with placements and supervision. Both of these elements appeared to suffer from a lack of formal structure; where some ACPs experienced a supportive clinical environment making their training experience ‘phenomenal’, others reported unsuitable supervision and having to identify their own supervisors and/ or placements. This lack of support was felt to have a serious negative impact on ACP training and well-being, which has been reported nationally and internationally [17, 31, 32]. It is recognised that a supportive environment is a healthy environment, aiding not only ACPs in their competency, role transition and job satisfaction but also helping to optimise quality patient care, recruitment and retention [13, 24]. Additionally, a disconnect between academic and clinical training was highlighted. This lack of ‘joined-up’ working between educators, healthcare staff and managers has been described previously with suggestions that it can impede the development of ACPs and their fulfilment of the role [22, 33].

The knowledge and experience already held by ACPs from their original professional training was perceived as both a strength and weakness. Whilst the ACPs felt they could provide improved holistic patient care and identify gaps in their own training, it influenced their approach to learning which was described as ‘bottom-up’ and ‘backwards’ compared to how junior doctors learnt. This had implications for confidence as ACPs often felt they could not adequately answer questions posed during training. Furthermore, if they were not given sufficient time to consolidate their new knowledge, this led to an additional drop in confidence and doubts about their competence. This was reported by MacLellan, Higgins and Levett-Jones (2017) [34] and has been referred to as Imposter Syndrome [35]. It links closely with role transition and identity which has been widely researched within the advanced practitioner community [12–14]. Increasing autonomy and responsibility is part of the transition for ACPs and whilst some of the ACPs in this study found this experience exciting, the majority conveyed mixed emotions including feeling stressed, pressured and uncertain. This was more prominent for those ACPs in areas where the role appeared to be less established and a lack of awareness among healthcare staff of the ACP role. For a smooth and successful transition, Barnes (2015) [12] identified a number of defining attributes including a shift from provider of care to prescriber of care, straddling two identities and mixed emotions. The experiences of our ACPs covered all of these attributes and suggest they have not experienced a smooth transitional journey.

Inconsistencies in the ACP training and lack of structure in relation to the clinical job role were discussed as contributing factors to role identity issues, which impacted their daily working lives. It appears the ACPs in our study are still experiencing the consequences of a role which was introduced without clear definition, standardisation, skills and scope [20], even though there has been significant development in recent years within advanced practice [5] of the ACP training. As a role introduced to work alongside doctors, nurses, pharmacists, and other healthcare professionals to deliver comprehensive and patient-centred care, ACPs play a pivotal role in fostering interprofessional collaboration within healthcare teams. However, with blurred definitions regarding the ACP role and responsibilities, it is unsurprising our ACPs reported a lack of understanding of their expertise and respect from their colleagues. Such barriers to interprofessional collaboration not only prevents ACPs from working to the full extent of their education and training [36] but impacts patients, on their outcomes and access to specialist care [21, 37, 38]. A review of 64 studies undertaken by Schot, Tummers and Noordegraaf (2020) of interprofessional collaboration among healthcare professionals described this as being multifaceted, and that for change to occur, individuals needs to work daily on tasks such as bridging gaps, negotiating overlaps and creating spaces [39].

There was agreement between the ACPs that accreditation of the role would help address some of the issues around role identity. The use of ‘advanced practice’ is widely applied within healthcare with little relationship to education level, often leading to confusion [18]. Accreditation would help protect the role by providing professional identity as well as providing more clarity to ACPs and those in the wider healthcare setting about the role and scope of practice [6, 17, 18]. It may also alieve fears of insecurity which were raised by the ACPs in relation to the future of the role. Improving and promoting knowledge brokering at both the individual and collective (system) levels would improve the transition process [40], whilst also encouraging change in an environment that is traditionally intransigent.

Although the ACPs reported challenges in their training and felt changes were necessary to ensure wider recognition of the ACP role, there was consensus among the ACPS that participated in this study that the training ‘boosted’ opportunities and allowed career progression whilst maintaining clinical responsibilities, an important factor to many of the ACPs in this study. Surprisingly, there was little discussion regarding the impact of the COVID-19 pandemic on ACP training, even though the focus groups took place during the pandemic. When it was discussed, it was generally in the context of placements and how they had been further limited.

This qualitative insight into the training experience of ACPs has highlighted that there are many challenges still to be overcome to ensure ACPs feel supported through their role transition journey and are recognised appropriately for their skills and experience in the healthcare workforce. These findings are not new [13, 14, 20, 22] but after the release of the 2017 HEE multi-professional framework for advanced clinical practice [5], it would be expected that there would have been more clarity and structure in the ACP training and role, benefitting ACPs, wider healthcare professionals and employers. Progress may improve as a result of the NMC review on regulation of advanced nursing practice that is due in the next 12 months [9], however, at the time of this study, the ACPs appeared to feel progress was slow and more work was needed.

### Strengths and limitations

The opinions and experiences provided in this study were from a group of ACPs, either during (> 1 year FTE) or post training, working in the South Yorkshire and Bassetlaw region. It is reasonable to suggest therefore that the results are not generalisable to other populations. Qualified and trainee ACPs were contacted about the study by email through the regional FACP-NEY who acted as gate keepers, as well as the study being advertised on social media platforms. It is assumed that this broad recruitment strategy helped to reach a wider population, although most respondents appeared to be as a result of the direct email. This approach may have introduced some bias but using a purposive sampling approach, participants from different specialties, professions and career stages were included. Information about the local ACP workforce such as size and individual characteristics was requested from the regional FACP-NEY but this was not provided thus an exact response rate cannot be calculated nor can any inferences be made regarding how representative the sample of ACPs were that participated in the study. The number of males that registered an interest in the study was low (three) and only one male participated in the focus groups; this is a limitation as there may be different perspectives and experiences of ACP training related to gender. Due to the COVID-19 pandemic focus groups had to be undertaken online. Adaptions were made to accommodate for this such as reducing the number of participants per focus group and creating time to build rapport [41]. One participant did experience technical issues, however using a digital approach did not appear to impede the participant-researcher interaction and compared favourably with traditional face to face focus groups [41, 42]. There is a risk that views from participants were oversimplified due to the limited number of ACPs involved in the focus groups but findings from this study appear to align with previously published literature [6, 17, 19, 21] providing some confidence in the results.

### Future work

This was a small exploratory study in a rapidly evolving field, providing insights on ACP training, role identity and competence at one point in time. ACPs did report differences in their experiences due to their specialty thus a much larger study would provide an opportunity to explore this further and allow for more in-depth comparisons. The multi-professional framework was relatively new when this study was undertaken and since its publication, there has been much development in the guidance and practice of ACPs including the Royal College of Emergency Care ACP training [43] and the merger of Health Education England with NHS England. It would be useful to explore what impact, if any, these developments may have had on ACPs and if similar issues around role identity and competence still exist.

## Conclusions

The ACP role is now integrated across many specialties both nationally and internationally, however challenges continue to persist in training, impacting on transition into the role. At a collective level, there remains a lack of structure and clarity around the ACP role, and individually ACPs appear to experience issues with supervision and support. This study has highlighted that the journey to advanced level practice is often turbulent, and changes are required to further embed the ACP training and role into the workplace. Ensuring ACPs have appropriate continuous support, allocated sufficient time to learn and practice, and wider recognition of the ACP role through accreditation would aid the training experience and a successful role transition.

### Electronic supplementary material

Below is the link to the electronic supplementary material.

Supplementary Material 1

## Data Availability

The datasets generated and analysed during the current study are not publicly available due to the participants privacy being compromised but are available from the corresponding author on reasonable request.

## Abbreviations

ACP: Advanced Clinical Practitioner
FACP-NEY: Faculty for Advanced Clinical Practice
HEE: Health Education England
NHS: National Health Service
